# Skull sinuses precluded extinct crocodile relatives from cetacean-style deep diving as they transitioned from land to sea

**DOI:** 10.1098/rsos.241272

**Published:** 2024-10-30

**Authors:** Mark T. Young, Julia A. Schwab, David Dufeau, Rachel A. Racicot, Thomas Cowgill, Charlotte I. W. Bowman, Lawrence M. Witmer, Yanina Herrera, Robert Higgins, Lindsay Zanno, Xu Xing, James Clark, Stephen L. Brusatte

**Affiliations:** ^1^ School of GeoSciences, Grant Institute, University of Edinburgh, Edinburgh EH9 3FE, UK; ^2^ LWL-Museum für Naturkunde, Sentruper Straße 285, Münster 48161, Germany; ^3^ School of Biological Sciences, Faculty of Environmental and Life Sciences, University of Southampton, Southampton, UK; ^4^ Department of Earth and Environmental Sciences, University of Manchester, Williamson Building, Oxford Road, Manchester M13 9PL, UK; ^5^ College of Osteopathic Medicine, Marian University, Indianapolis, IN, USA; ^6^ Department of Messel Research and Mammalogy, Senckenberg Research Institute and Natural History Museum, Senckenberganlage 25, Frankfurt am Main 60325, Germany; ^7^ Department of Biomedical Sciences, Heritage College of Osteopathic Medicine, Ohio University, Athens, OH, USA; ^8^ CONICET, División Paleontología Vertebrados, Unidades de Investigación Anexo Museo, Facultad de Ciencias Naturales y Museo, UNLP, La Plata, Argentina; ^9^ Paleontology, North Carolina Museum of Natural Sciences, Raleigh, NC, USA; ^10^ Department of Biological Sciences, North Carolina State University, 100 Brooks Avenue, Raleigh, NC 27607, USA; ^11^ Centre for Vertebrate Evolutionary Biology, Yunnan University, Kunming 650031, People’s Republic of China; ^12^ Institute of Vertebrate Paleontology & Paleoanthropology, Chinese Academy of Sciences, Beijing 100044, People’s Republic of China; ^13^ Department of Biological Sciences, George Washington University, Washington, DC, USA; ^14^ Department of Natural Sciences, National Museums Scotland, Chambers Street, Edinburgh, UK

**Keywords:** Mesozoic, multivariate analyses, morphospace, convergence, sinuses, macroevolution, marine tetrapods

## Abstract

During major evolutionary transitions, groups develop radically new body plans and radiate into new habitats. A classic example is cetaceans which evolved from terrestrial ancestors to become pelagic swimmers. In doing so, they altered their air-filled sinuses, transitioning some of these spaces to allow for fluctuations in air capacity and storage via soft tissue borders. Other tetrapods independently underwent land-to-sea transitions, but it is unclear if they similarly changed their sinuses. We use computed tomography to study sinus changes in thalattosuchian crocodylomorphs that transformed from land-bound ancestors to become the only known aquatic swimming archosaurs. We find that thalattosuchian braincase sinuses reduced over their transition, similar to cetaceans, but their snout sinuses counterintuitively expanded, distinct from cetaceans, and that both trends were underpinned by high evolutionary rates. We hypothesize that aquatic thalattosuchians were ill suited to deep diving by their snout sinuses, which seem to have remained large to help drain their unusual salt glands. Thus, although convergent in general terms, thalattosuchians and cetaceans were subject to different constraints that shaped their transitions to water. Thalattosuchians attained a stage similar to less pelagic transitional forms in the cetacean lineage (late protocetid-basilosaurid) but did not become further specialized for ocean life.

## 1. Introduction

Evolutionary radiations often occur after a radical shift in body plan, when a group of organisms undergoes a major evolutionary transition that allows them to explore new habitats. A textbook example is secondarily aquatic tetrapods: over the last ~300 million years of evolution, over 30 groups of land-living vertebrates have refashioned their bodies to move into the water (e.g. [1–9]). In doing so, these disparate groups would have experienced common selection pressures, such as moving and feeding in a more viscous and dense three-dimensional medium compared to air, which resulted in convergences of skeletal morphology, physiology and behaviour.

Cetaceans (whales and dolphins) are the classic case of a secondarily aquatic group: they are among the most recent tetrapods to go from land to sea, and their transformation is captured by a rich fossil record of transitional species. Among their many adaptations to the pelagic realm are profoundly transformed air sinuses within their skulls [10–12]. Cetaceans had a curious mix of sinus morphologies, reducing the number and volume of bone-enclosed sinuses compared to their terrestrial ancestors, but also evolving expansive extracranial pterygoid sinuses and air sacs (e.g. [10–14]). The reduction in bone-enclosed sinuses is thought to have alleviated depth-related increases in hydrodynamic pressure within the skull and the respiratory system itself, enabling deeper dives, while the expansive extracranial sinuses are thought to aid sound production and reflection (facilitating bidirectional hearing) (see [10–12,15–22]). It is unclear, however, if other groups of pelagic tetrapods modified their cranial sinuses in similar ways.

**Figure 1.**
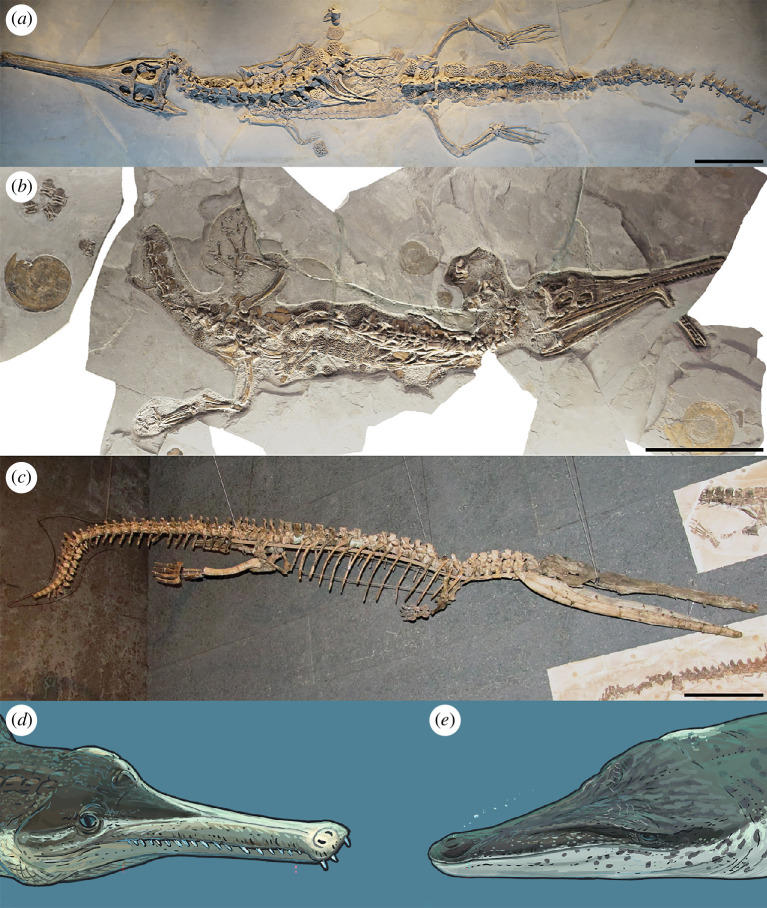
The variation in thalattosuchian body plans, with semi-aquatic thalattosuchians (*a,b*) compared to a fully aquatic metriorhynchid (*c*). (*a*) The machimosaurid teleosauroid *Macrospondylus bollensis* (referred specimen NMW 13445); (*b*) the early diverging metriorhynchoid *Pelagosaurus typus* (referred specimen FWD 0784); (*c*) the metriorhynchid *Thalattosuchus superciliosus* (referred specimen GPIT-PV-31379). Life reconstructions of (*d*) a teleosauroid and (*e*) a metriorhynchid made by Joschua Knüppe. Scale bars, (*a*,*b*) 20 cm and (*c*) 30 cm.

Answering this question is difficult, as few other land-to-sea shifts in tetrapods are documented by a series of well-preserved transitional fossils. One group that fulfils this criterion is the thalattosuchians (figure 1), a clade of extinct crocodile relatives (crocodylomorphs) that lived during the Jurassic and Cretaceous periods (*ca* 199−121 million years ago) (see [23–26]). Thalattosuchians evolved from terrestrial ancestors and include two major subclades, the first being the semi-aquatic teleosauroids (figure 1*a*), a freshwater and nearshore group that superficially resembled extant long-snouted crocodylians like gharials (e.g. [27–32]). The second subclade is the metriorhynchoids (figure 1*b,c*), which include the highly derived metriorhynchids—the only purely pelagic swimming archosaurs in Earth history (e.g. [27,28,31,33–37]). Metriorhynchid flippers, tail flukes and streamlined bodies have generated frequent comparisons with cetaceans (see [37] for exceptional soft tissue preservation in Metriorhynchidae), and recent work shows that their inner ear sensory system also became compact and smaller as they relocated into the water, just as cetaceans independently evolved millions of years later [38]. Yet, the extent of convergence between thalattosuchians and cetaceans remains to be fully explored. It has been noted that some thalattosuchians had reduced air sinuses in their braincases [39–44], but other studies have identified large snout sinuses in pelagic metriorhynchids [45–47]. These findings raise the question: did thalattosuchians reduce their sinuses in a manner similar to cetaceans, or not? And what might the answer reveal about the habits and habitats of thalattosuchians, and how their transition compared to that undertaken by cetaceans some 100 million years later?

**Figure 2.**
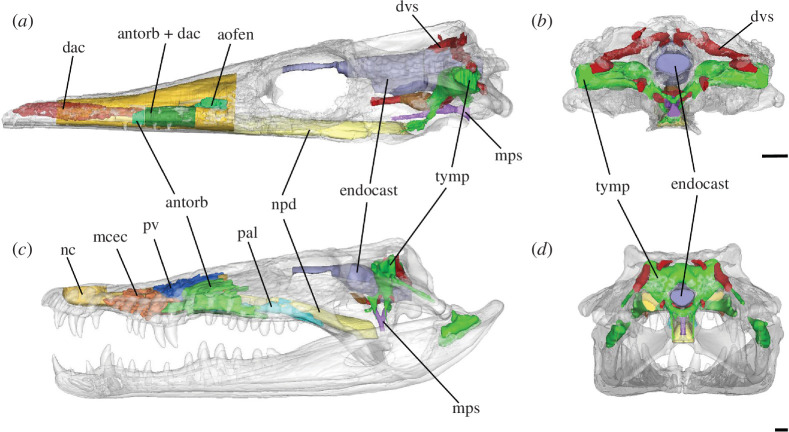
Transparent skulls of (*a,b*) *Pelagosaurus typus* (NHMUK PV OR 32599) and (*c,d*) *Crocodylus rhombifer* (MNB AB50.0171) showing the cranial sinus systems. Abbreviations: antorb, antorbital sinus; aofen, antorbital fenestra; dac, dorsal alveolar canal; dvs, dural venous sinus; mcec, maxillary cecal recess; mps, median pharyngeal sinus; nc, nasal cavity; npd, nasopharyngeal ducts; pal, palatine sinus; pv, postvestibular sinus; tymp, paratympanic sinuses. Scale bars, 1 mm. Note that the separation between the antorbital sinus and the dorsal alveolar canal in *Pelagosaurus* is difficult to distinguish (see [47]).

Modern crocodylians have highly pneumatic skulls, excavated by two major sinus systems (figure 2). The paratympanic (‘braincase’) sinuses are air-filled epithelial outgrowths of the pharynx that form the middle ear cavity and infiltrate every bone of the braincase in modern crocodylian species (e.g. [48–51]). The paranasal (‘snout’) sinuses are epithelial outgrowths of the nasal cavity that infiltrate the bones of the snout and secondary palate [52–54]. The oldest fossil crocodylomorphs (the terrestrial ‘sphenosuchians’) underwent a general increase in skull pneumaticity (particularly in the palatal bones, quadrate and dorsal half of the braincase), coinciding with the development of an akinetic skull with a bony palate and tightly sutured braincase [49,55–59]. Other early diverging crocodyliforms (such as protosuchians) shared with extant species a sutured and pneumatized braincase [60–62], whereas later diverging crocodyliforms with a fully developed bony palate (notosuchians and neosuchians) also had extensive braincase and snout sinuses (e.g. [63–66]). Thalattosuchians, on the other hand, were apparently outliers: they had an akinetic skull that seems to have been poorly pneumatized, at least in the braincase (figure 2), but their sinus systems have never been subjected to detailed analysis in a statistical and phylogenetic context.

**Figure 3.**
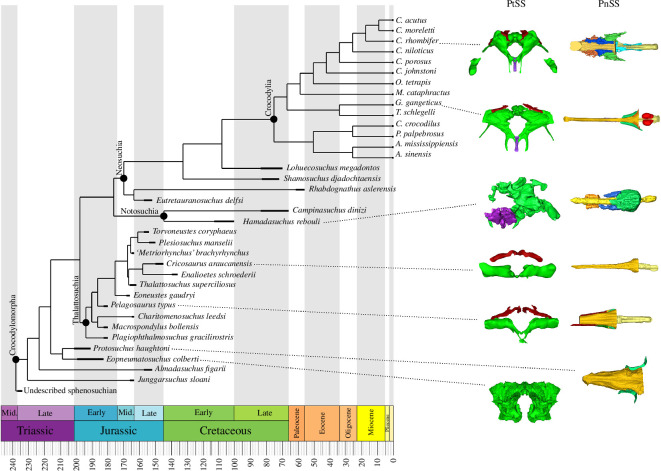
Simplified time-scaled phylogeny showing the paratympanic (PtSS, shown in occipital view) and paranasal sinus (PnSS, shown in dorsal view) systems of key extinct and extant crocodylomorph taxa used in our study. The sinus systems seen in Thalattosuchia are ‘simplified’ compared to other crocodyliforms, the PtSS sinuses in notosuchians and protosuchids are more complex, while PnSS sinuses in notosuchians and extant crocodylians are more elaborate. Green represents the pharyngotympanic sinus system of the PtSS and the antorbital sinus of the PnSS. The blue and orange are accessory PnSS sinuses. Yellow represents the nasal cavity, with gold being the nasopharyngeal duct and bright orange the gharial pterygoid bullae. Red presents primarily vascular canals. Models are not to scale.

Here we study evolutionary trends in the paratympanic and paranasal sinuses in Crocodylomorpha (figure 3). We used computed tomography (CT) scans to identify the sinuses and determine their size and shape, in 22 species of fossil crocodylomorph (including 11 thalattosuchians) and 14 modern crocodylians. This was supplemented by three well-described species from the literature (see electronic supplementary material, appendix S1). We then used character optimizations on a series of phylogenetic topologies generated from Young *et al*. [25], multivariate analyses and phylogenetically informed statistical analyses to test whether there were changes in the sinuses of thalattosuchians as they went from land to shallow water to the open ocean, and how these compared to cetaceans. This allows us to ask whether there may be a general ‘rule’ of how secondarily aquatic tetrapods alter their sinuses, or whether there were constraints or nuances of their biology that forced thalattosuchians and cetaceans down different paths.

## 2. Methods

### 2.1. Phylogenetic framework and character optimization

There is a long-standing debate about the position of Thalattosuchia in the crocodylomorph family tree [67–69], which may affect determination of character polarity at the base of the clade and thus our understanding of sinus evolution. The main competing hypotheses are that Thalattosuchia is: (i) the sister taxon to Crocodyliformes, (ii) an early diverging member of Mesoeucrocodylia, (iii) an early diverging member of Neosuchia, and (iv) a member of Tethysuchia (see [25] for more details). Young *et al.* [25] performed a series of phylogenetic analyses to explore positional hypotheses of Thalattosuchia within Crocodylomorpha. To do so, they ran a series of topological constraint analyses under both equal and extended implied character weighting schemes. These analyses yielded eight distinct topologies. Although hypothesis two (= early diverging member of Mesoeucrocodylia) was recovered as the most parsimonious under both the equal weighting and extended implied weighting analyses, Young *et al.* [25] could not statistically discriminate between hypothesis two and the alternative topologies derived from topological constraint analyses. As such we utilized all eight distinct topologies in our phylogenetic comparative analyses. Herein we use the systematization and nomenclature for Thalattosuchia that were outlined by Johnson *et al.* [32], Young *et al.* [25] and Sachs *et al.* [70].

### 2.2. Computed tomography dataset

To create the sinus character dataset (see electronic supplementary material, appendix S1), we scored skull specimens based on either first-hand observation of CT scans or well-described species from the literature (see electronic supplementary material, appendix S1, for the sources of all taxa). Some of the scans were collected during the tenure of this project (those scanned at the University of Southampton), while others were sourced from the online databases MorphoSource (https://www.morphosource.org/) and DigiMorph (http://digimorph.org/). Sinus cavities were segmented from the scans using Materialise Mimics versions 19.0–21.0, using the 3D livewire and lasso tools.

Our sample size is limited by the need to score as many sinus characters as possible for our quantitative analyses (below). As such, the paratympanic and paranasal datasets have different samples. The paratympanic dataset has 35 species: three early diverging crocodylomorphs (undescribed sphenosuchian, *Junggarsuchus sloani* and *Almadasuchus figarii*) two early diverging crocodyliforms (*Protosuchus haughtoni* and *Eopneumatosuchus colberti*), two notosuchians (cf. *Hamadasuchus rebouli* and *Campinasuchus dinizi*), four fossil neosuchians (*Eutretauranosuchus delfsi*, *Rhabdognathus aslerensis*, *Shamosuchus djadochtaensis* and *Lohuecosuchus megadontos*), two extant gavialids (*Gavialis gangeticus* and *Tomistoma schlegelii*), eight extant crocodylids (*Osteolaemus tetraspis*, *Mecistops cataphractus*, *Crocodylus acutus*, *Croc. johnstoni*, *Croc. moreletii*, *Croc. niloticus*, *Croc. porosus* and *Croc. rhombifer*), four extant alligatorids (*Alligator mississippiensis*, *Al. sinensis*, *Caiman crocodilus* and *Paleosuchus palpebrosus*), four semi-aquatic thalattosuchians (*Plagiophthalmosuchus gracilirostris*, *Macrospondylus bollensis*, *Charitomenosuchus leedsi* and *Pelagosaurus typus*) and six pelagic metriorhynchids (*Enalioetes schroederi*, *Cricosaurus araucanensis*, *Thalattosuchus superciliosus*, ‘*Metriorhynchus*’ cf. *brachyrhynchus*, *Torvoneustes coryphaeus* and *Plesiosuchus manselii*). Summarized in electronic supplementary material, appendix S1, table S1.4).

The paranasal dataset has 26 species: one early diverging crocodylomorph (*Junggarsuchus sloani*), one early diverging crocodyliform (*Protosuchus haughtoni*), two notosuchians (cf. *Hamadasuchus rebouli* and *Campinasuchus dinizi*), one fossil neosuchian (*Lohuecosuchus megadontos*), two extant gavialids (*Gavialis gangeticus* and *Tomistoma schlegelii*), eight extant crocodylids (*Osteolaemus tetraspis*, *Mecistops cataphractus*, *Crocodylus acutus*, *Croc. johnstoni*, *Croc. moreletii, Croc. niloticus*, *Croc. porosus* and *Croc. rhombifer*), four extant alligatorids (*Alligator mississippiensis*, *Al. sinensis*, *Caiman crocodilus* and *Paleosuchus palpebrosus*), four semi-aquatic thalattosuchians (*Plagiophthalmosuchus gracilirostris*, *M. bollensis*, *P. typus* and *Eoneustes gaudryi*), and three pelagic metriorhynchids (*Enalioetes schroederi*, *Cricosaurus araucanensis* and *T. superciliosus*). Summarized in electronic supplementary material, table S1.5, appendix S1).

### 2.3. Sinus character dataset

The terminology used herein for the paratympanic sinus system follows Dufeau & Witmer [50] and for the paranasal sinus system we follow Witmer [53]. We compiled two datasets of sinus system characters (see electronic supplementary material, appendix S1). We had 24 paratympanic (‘braincase’) sinus characters, spanning both the median pharyngeal sinus system and the pharyngotympanic sinus system. Many characters were scored based on the presence/absence of internal cavities based on examining the CT scans and relevant literature. Other characters were more complex (such as the morphology of internal structures) but were scored using the same data sources. For the external foramina, characters were scored on observation of the specimens and CT scans.

For the paranasal (‘snout’) sinus dataset, we had 15 characters. This character suite spanned the various internal cavities formed by infiltrations from the nasal cavity and nasopharyngeal duct, as well as the external morphology of the antorbital fenestrae. As with the paratympanic dataset, internal cavities were scored based on observation of the CT scans, while the external antorbital fenestrae morphologies were scored based on observation of the specimens.

### 2.4. Ecological categories

To investigate whether there is an ecological signal in the sinus datasets we used three different sets of ecomorphological groupings: the terrestrial-freshwater-marine categorization from Wilberg *et al.* [31]; the terrestrial-semiaquatic-pelagic categorization from Schwab *et al.* [38]; and a simpler categorization of terrestrial-aquatic similar to Bronzati *et al.*’s [71] swimming capability category. The categorization protocols follow from the papers that originated them, with the general ‘aquatic’ grouping including all species not in the terrestrial grouping (see electronic supplementary material, table S1.6, appendix S1).

### 2.5. Multivariate analyses

We conducted the multivariate and phylogenetic comparative methods in R version 4.4.0 ‘Puppy Cup’ [72]. The two sinus character datasets were analysed using principal coordinates analysis (PCo) in the R package Claddis 0.6.3 [73], where we generated ordination spaces for visualization. The PCo axes that cumulatively accounted for 95% of the variation were used for subsequent ordination analyses and clade separation multivariate statistics. We used PERMANOVA to test whether different habitat and the thalattosuchian and metriorhynchid clade groups were significantly separated from each other in the PCo morphospace using the pairwiseAdonis() function in the R package vegan 2.6-4 [74]. We also performed a canonical variate analysis in the R package Morpho 2.12 [75] to test the ability of the PCo scores to assign individuals to the *a priori* ecological and clade categories.

### 2.6. Phylogenetic comparative methods

Given the uncertainty of the position of Thalattosuchia within Crocodylomorpha, we ran all comparative phylogenetic methods for each topology (i.e. eight times). This was to gauge whether phylogenetic position impacted our interpretation of sinus evolution. We pruned the phylogenetic topologies to match the taxon sampling for the paratympanic and paranasal sinus datasets (see electronic supplementary material, appendix S2, S4). The time-scaled chronograms were created using R package strap 1.6-0 [76] using the ‘equal’ method set out by Brusatte *et al.* [77].

We tested for phylogenetic signal in the PCo scores with Pagel’s lambda (λ), using the R package phytools 2.1-1 [78]. A λ value close to 0.0 means that the covariance between species does not match that expected under Brownian evolution, whereas a λ close to 1.0 means that the covariance between species is close to the covariances expected under Brownian evolution. Only those correlations with a Bonferroni corrected alpha-value equal to or below 0.0167 were accepted as statistically significant (see electronic supplementary material, appendix S2). We tested for partitioning of variance with Blomberg’s K using the R package phytools 2.1-1 [78]. When K is greater than 1 variance tends to be partitioned between clades, whereas when K is less than 1 variance is partitioned within clades. Only those correlations with a Bonferroni corrected alpha-value equal to or below 0.0167 were accepted as statistically significant (see electronic supplementary material, appendix S2).

We tested whether there was a correlation between the paratympanic and paranasal sinus PCo scores with the *a priori* habitat groups and the Thalattosuchia clade group, using phylogenetic generalized least square regression in the R package nlme 3.1-164 [79]. The fit of each model was assessed with maximum likelihood and the best supported model was determined by the lowest AIC score. Only those correlations with a Bonferroni corrected alpha-value equal to or below 0.007 were accepted as statistically significant (see electronic supplementary material, appendix S2).

We optimized the first three PCo scores as a continuous variable onto the phylogeny, to predict ancestral states for major clades and assess evolutionary trends (electronic supplementary material, appendix S2). The optimizations were performed using maximum likelihood and the fastAnc() function in the R package phytools 2.1-1 [78].

We fitted seven standard models of trait evolution to the first three paratympanic and paranasal sinus PCo scores on the phylogeny: Brownian motion, Ornstein–Uhlenbeck (OU), early burst, Brownian motion with a directional trend, Pagel’s Lambda, Pagel’s Kappa, and Pagel’s Delta. The fit of each model was assessed with maximum likelihood and the best-supported model was determined by the lowest AICc score, using the R package geiger 2.0.11 [80].

To determine whether any branches along the various phylogenies had higher rates of morphological evolution we used the test_rates function in the R package Claddis 0.6.3 [73]. The five branches with the lowest AICc scores were chosen for each of the six topologies (for both the paratympanic and paranasal sinus datasets) to assess whether the position of Thalattosuchia impacts sinus evolutionary rates. Finally, we visualized the rates of evolution using the phenogram function in the R package phytools 2.1-1 [78].

## 3. Results

### 3.1. Phylogenetic character optimization

While there is disagreement about the position of Thalattosuchia within Crocodylomorpha [67–69], character optimization on the eight phylogenetic topologies reveals a consistent pattern no matter the placement of Thalattosuchia. Braincase sinuses reduced across thalattosuchian evolution, and as the clade became more aquatic. At the base of Thalattosuchia, the proötic and quadrate diverticula were lost or vestigial, quadrate pneumatic foramina were lost, otoccipital diverticula were limited dorsolaterally and the intertympanic diverticula absent and the internal sinus chambers were openly contiguous. The fully aquatic (= pelagic) metriorhynchids continued this trend, with loss of the pharyngotympanic tubes and basioccipital diverticula, and further reduction of the otoccipital diverticula in volume. Paranasal sinuses of the snout, on the other hand, expanded. At the base of Thalattosuchia the paranasal sinuses were a simple internal tube; aquatic metriorhynchids, however, had dorsoventrally deepened nasopharyngeal ducts, expanded posterior antorbital cavities, laterally enclosed antorbital cavities and extracranial suborbital diverticula.

### 3.2. Principal coordinates analysis

**Figure 4.**
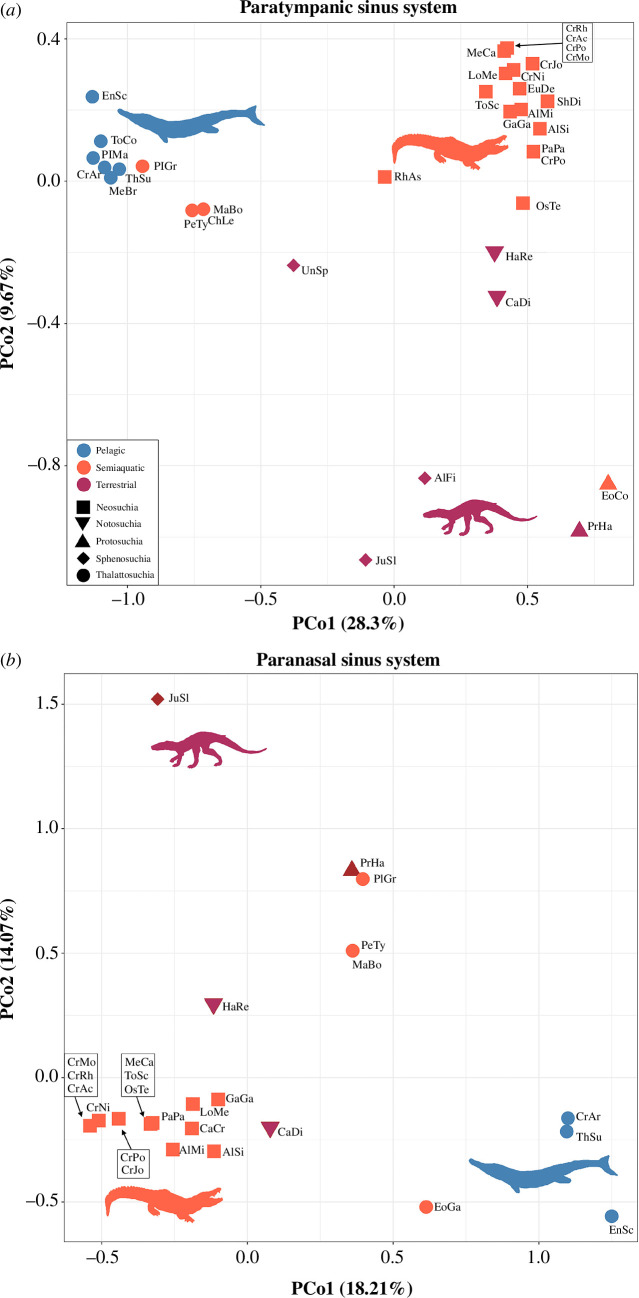
Shape morphospace showing distribution of extinct and extant crocodylomorphs and their habitat preferences. (*a*) PCo1 versus PCo2 of the paratympanic sinus system; (*b*) PCo1 versus PCo2 of the paranasal sinus system. These morphospace plots demonstrate that the paratympanic sinus system of Thalattosuchia was distinct from that of all other crocodylomorphs, whereas the paranasal sinus system of Metriorhynchidae was distinct. See electronic supplementary material, appendix 1, tables S1.2 and S1.3, for the specimen key. The silhouette images are taken from Schwab *et al.* [38].

PCo of our paratympanic sinus dataset, which condenses information from all of the sinuses into a more manageable set of variables, ordinated a morphospace in which the first two axes describe 37.97% of the total variance (figure 4*a*). PCo1 explains 28.30% of the variance and separates Thalattosuchia from all other taxa. Pelagic metriorhynchids have the most negative PCo1 scores, whereas protosuchians (early diverging crocodyliforms) have the most positive scores. PCo2 explains 9.67% of the variance and separates the majority of crocodylomorphs from two derived ‘sphenosuchians’ and protosuchians (i.e. the species with the most heavily pneumatized ventral braincases). The two PCo axes together show: (i) the semi-aquatic thalattosuchians do not cluster with the semi-aquatic neosuchians, (ii) Thalattosuchia is distinct from all other crocodylomorphs, and (iii) pelagic metriorhynchids are the most extreme in their sinus morphologies (on PCo1).

The PCo of our paranasal sinus dataset ordinates a morphospace in which the first two axes describe 32.28% of the total variance (figure 4*b*). PCo1 explains 18.21% of the variance and separates Metriorhynchidae from all other taxa. The pelagic metriorhynchids have the most positive PCo1 scores, whereas extant crocodylians like *Crocodylus* have the most negative scores. PCo2 explains 14.07% of the variance and does not clearly delimit clades or habitat groups. The two PCo axes together show: (i) the semi-aquatic thalattosuchians do not cluster with the semi-aquatic neosuchians, (ii) the non-neothalattosuchian thalattosuchian *Plagiophthalmosuchus* is not distinct from all other crocodylomorphs—instead it is close in position to *Protosuchus*, (iii) the early diverging metriorhynchoid *Eoneustes* is the closest in position to metriorhynchids, and (iv) the pelagic metriorhynchids are the most extreme in their sinus morphologies (PCo1 and PCo2).

**Figure 5.**
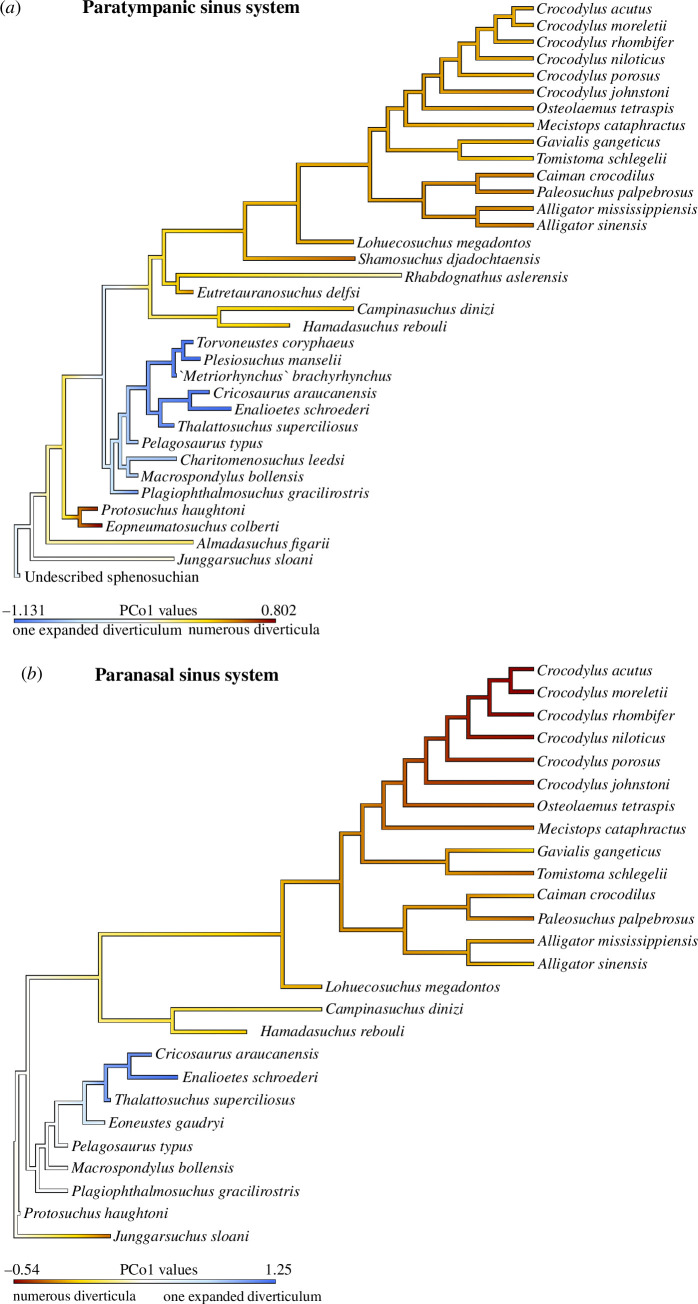
PCo1 scores plotted on a phylogeny to show pneumaticity in crocodylomorph taxa. (*a*) Paratympanic sinus system (where blue indicates low pneumaticity and red indicates high pneumaticity; (*b*) paranasal sinus system (where blue indicates a single paranasal sinus that is greatly expanded and red indicates numerous complex paranasal diverticula). This demonstrates the paratympanic sinus system of Thalattosuchia was distinct from that of all other crocodylomorphs, whereas only the paranasal sinus system of Metriorhynchidae was distinct.

In sum, the PCo analyses of the two sinus system datasets show four key results. First, semi-aquatic thalattosuchians never cluster with semi-aquatic neosuchians (figure 4), so despite similar habitats and lifestyles they had distinctive paratympanic and paranasal sinus systems. Second, thalattosuchians had a distinct paratympanic sinus morphotype among crocodylomorphs (figures 4 and 5), which, based on character optimization, we can identify as an extensive loss of bone-enclosed sinuses that were present in their ancestors. Third, early diverging thalattosuchians like *Plagiophthalmosuchus* were similar in paranasal sinus morphospace to early crocodylomorphs like *Protosuchus* (figure 4), meaning that thalattosuchians as a whole did not have a distinct paranasal sinus morphotype among crocodylomorphs (unlike their unusual paratympanic system). Fourth, the pelagic metriorhynchids are outliers in both the paratympanic and paranasal morphospaces, even compared to semi-aquatic thalattosuchians, which character optimization reveals is due to their extremely reduced paratympanic sinuses and enlarged paranasal sinuses (figures 4 and 5).

### 3.3. Multivariate statistical tests

Statistical tests of morphospace occupation (PERMANOVA) for both the paratympanic and paranasal datasets support the observation that habitat and taxonomic groups form clusters in morphospace (see electronic supplementary material, appendix S2). In different tests, terrestrial, semi-aquatic and pelagic species (*sensu* [38]) are statistically distinct from one another, as are terrestrial, freshwater and marine species (*sensu* [31]), and simply terrestrial and ‘aquatic’ species (*sensu* [71]). This is also the case when thalattosuchians are compared to non-thalattosuchians, and metriorhynchids to non-metriorhynchids. The only exceptions are two habitat comparisons for the paranasal dataset (terrestrial-pelagic and terrestrial-marine) that fail to reach significance because of small sample size and our use of a Bonferroni-corrected alpha-value.

Canonical variates analyses (CVA) on the PCo scores further demonstrate that the paratympanic and paranasal sinus datasets have both habitat and taxonomic signals. When all crocodylomorphs are placed into habitat groupings predetermined from osteological and environmental evidence, and individual taxa are then iteratively treated as having an unknown habitat, the CVA classifies them into the correct habitat 100% of the time (electronic supplementary material, appendix S2). The same is true when crocodylomorphs are placed into predetermined clade groupings (see electronic supplementary material, appendix S2).

### 3.4. Phylogenetic comparative methods

Due to the relationship between clade membership and PCo scores, we further explored the relationship between phylogeny and morphology using phylogenetic comparative methods. Regardless of where Thalattosuchia is placed on the crocodylomorph tree, Pagel’s lambda shows that there is a strong and significant phylogenetic signal for the first three PCo axes in both sinus datasets (electronic supplementary material, appendix S2). Blomberg’s K corroborates that there is usually (but not always) considerable phylogenetic signal on these PCo axes (see electronic supplementary material, appendix S2).

Given the presence of a phylogenetic signal in our datasets, we used phylogenetic regressions (pGLS) to further explore relationships between sinus morphology, habitat and clade membership. Our key finding is that, even with phylogeny considered, sinus morphology is often still statistically correlated with habitat and taxonomic groups, especially for the first principal coordinate axis (see §7 in electronic supplementary material, appendix S2, for a summation). The degree of significance varies, however, depending on which phylogenetic hypothesis is used, and may be dampened by small sample size and our Bonferroni corrections (see electronic supplementary material, appendix S2). Overall, the pGLS results suggest that sinus morphology is most strongly correlated with clade membership (Thalattosuchia and/or Metriorhynchidae), and that their aberrant morphologies may be driving the larger habitat clusters that are statistically distinct in other analyses above but not always when phylogeny is accounted for here. Given the strong correlation between clade membership (Thalattosuchia and Metriorhynchidae) and sinus morphology, we caution against overinterpreting habitat relationships. Future analyses with a broader sample of Mesozoic crocodylomorphs are needed to determine whether the habitat signal found herein is genuine or an artefact due to the high number of thalattosuchians.

To explore the tempo and mode of sinus change during thalattosuchian evolution, we fitted seven standard models of trait evolution to the first three PCo axis scores for both sinus datasets. Regardless of phylogenetic hypothesis, an early burst model was best supported for the first three PCo axes for the paratympanic sinus system (electronic supplementary material, appendix S2). Regardless of phylogenetic hypothesis, for the paranasal sinus system, an early burst model was best supported for PCo1 and PCo2, while for PCo3 Pagel’s Kappa model (evolutionary trait change occurring after cladogenesis, i.e. the internal nodes) was best supported (see electronic supplementary material, appendix S2). Given that the early burst models are repeatedly supported for five of the six most important PCo axes, this suggests there was rapid trait evolution occurring early in crocodylomorph history. This supports the previously noted expansion of the palatal and dorsal braincase sinus diverticula as the akinetic skull evolved [53–56,62,63] and the marked reduction of braincase pneumaticity at the base of Thalattosuchia [39–41,43].

To further explore the tempo of sinus changes, we analysed evolutionary rates. When the phylogeny is plotted such that the *x*-axis is scaled to time and the *y*-axis to PCo1 score, metriorhynchids/thalattosuchians are visually shown to have higher rates of PCo1 evolution than other crocodylomorphs for both sinus systems (electronic supplementary material, appendix S2). There is a consistent general pattern of quantitative evolutionary rates along the phylogeny for the two sinus datasets, with the branches leading to Thalattosuchia, Metriorhynchidae, or closely related groups having the highest rates for a given phylogenetic hypothesis. For the paratympanic dataset, the thalattosuchian branch almost always exhibited the highest rate (for seven out of the eight analyses), with Metriorhynchidae not far behind (being the third, fourth or fifth highest-rated branch; see electronic supplementary material, appendix S2). For the paranasal dataset, Metriorhynchidae always had the highest rate. Taken together, these results support the hypothesis that there was an evolutionary trend of increasingly specialized cranial sinus morphotypes in thalattosuchians, culminating in the most aberrant morphologies in pelagic species (Metriorhynchidae), which involved relatively rapid rates of change compared to the background. They also indicate that the paratympanic sinus system underwent a dramatic shift at the base of Thalattosuchia.

## 4. Discussion

### 4.1. Thalattosuchian sinus reduction

**Figure 6.**
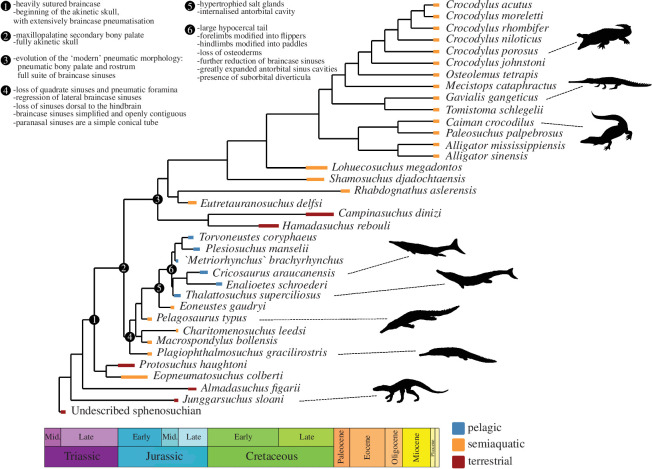
Aquatic adaptations for Metriorhynchidae, and the major trends in the formation of the akinetic skull and cranial pneumaticity plotted on a time-scaled crocodylomorph phylogeny (= mesoeucrocodylian hypothesis). This shows that the major paratympanic changes occurred at the base of Thalattosuchia, long before the transition to a pelagic swimming ecology, and that the evolution of hypertrophied salt glands evolved prior to the expanded paranasal sinuses. Node 1 = Crocodyliformes, node 2 = Mesoeucrocodylia, node 3 = Metasuchia, node 4 = Thalattosuchia, node 5 = *Eoneustes* + Metriorhynchidae and node 6 = Metriorhynchidae. Four of the silhouette images were taken from Schwab *et al.* [38], the two metriorhynchids, and the silhouettes adjacent to *Plagiophthalmosuchus gracilirostris* and *Junggarsuchus sloani*. The other silhouettes were taken from PhyloPics.org. The silhouette adjacent to *Crocodylus porosus* is by Steven Traver (CC0 1.0 Universal Public Domain Dedication), the one adjacent to *Caiman crocodilus* is by B. Kimmel (Public Domain Mark 1.0), the one adjacent to *Gavialis gangeticus* is by Evan Boucher (Public Domain Mark 1.0), the one adjacent to *Pelagosaurus typus* is by Nobu Tamura and vectorized by T. Michael Keesey (Attribution 3.0 Unported license https://creativecommons.org/licenses/by/3.0/). No changes were made to any of these silhouettes.

We find that Mesozoic thalattosuchians evolved a unique suite of skull sinus morphologies radically distinct from all other crocodylomorphs, and these developed through high rates of evolution and culminated in the exceptionally transformed sinuses of the only fully aquatic (= pelagic) swimming archosaurs that ever lived, the metriorhynchids. Regardless of their placement in the crocodylomorph phylogeny, thalattosuchians experienced a dramatic reduction in braincase pneumaticity compared to their ancestors. In all thalattosuchians, these modifications included lost or vestigial proötic and quadrate diverticula, loss of quadrate pneumatic foramina, dorsolaterally limited otoccipital diverticula and an overall simplification of the braincase sinuses with the various internal structures being openly contiguous. Metriorhynchids, however, went a step further and lost the pharyngotympanic tubes and basioccipital diverticula, lacked a basisphenoid diverticulum distinct from the median pharyngeal sinus, and further reduced the otoccipital diverticula. Furthermore, relative to other thalattosuchians and all other crocodylomorphs, metriorhynchids had the most aberrant snout sinus systems, as they evolved extracranial suborbital diverticula, greatly expanded posterior antorbital cavities and dorsoventrally deepened nasopharyngeal ducts (figure 6).

Reduction in braincase pneumaticity at the base of Thalattosuchia is intriguing. Like modern crocodylians, thalattosuchians had an akinetic skull with a bony secondary palate and a well-sutured braincase, but unlike modern crocodylians and their ancestors, thalattosuchians lacked the extensive opportunistic infiltration of the skull bones by pneumatic epithelial diverticula [39–44]. It is worth noting however that thalattosuchian skulls have several skull roof and braincase characteristics that make them peculiar for crocodyliforms (which have been suggested to be evidence that thalattosuchians lie outside of Crocodyliformes), including the absence of the ‘skull table’ morphology and reduced sutural contact between the quadrate and the braincase (e.g. the loss of the quadrate-laterosphenoid contact and exposure of the proötic in dorsal view) (see [24,25] for recent overviews). Currently, it is unknown if these skull characteristics reflect their non-crocodyliform origins or are a consequence of paedomorphosis (including in some instances delayed ossification) that has been hypothesized to have reshaped the thalattosuchian, and metriorhynchid in particular, skeleton [45,81–83].

Amongst extant diving birds and mammals, there is a trend of reduced skull pneumaticity compared to their relatives that either are more terrestrial or do not engage in pursuit diving [84,85]. Modern pursuit diving birds (e.g. diving petrels, loons, grebes and penguins) also have reduced postcranial pneumaticity [84]. Curiously, reduction of skull sinuses occurs not only in deep-divers, as this trend is seen in both polar bears and river otters—species that dive only to shallower depths of 10−15 m [85]. The loss of the intertympanic diverticulum is a ‘classic’ characteristic of thalattosuchians, as it marks the absence of one interaural pathway (the various paratympanic sinuses acoustically couple the two middle ears in crocodylians and birds, improving directional hearing of airborne sounds; see [50,86–89]). During the Eocene, penguins also began a marked reduction in skull pneumaticity, including the loss of the intertympanic diverticula [90]. A similar trend is observed in semi-aquatic extinct cetaceans, which show a decline in skull sinus volume [13,14]. Reduction of skull sinuses, therefore, appears to be a theme for tetrapods that engage in pursuit hunting behaviour within the water column.

### 4.2. Why did braincase sinuses reduce in thalattosuchians?

We propose three hypotheses as to why thalattosuchians had reduced cranial pneumaticity but note that these hypotheses are not mutually exclusive. Although specific to thalattosuchians, they are potentially applicable to other secondarily aquatic tetrapods that underwent similar changes.

*One*: reduced pneumaticity may have helped to increase skull density, thus decreasing buoyancy and improving diving performance, as has been demonstrated in modern diving birds [84]. Buoyancy may have been a particular problem for early thalattosuchians, as they had the proportionally largest heads relative to body length of all crocodylomorphs [91,92]. Although in the *Eoneustes*+Metriorhynchidae subclade, the skull bones became highly cancellous, which has been hypothesized to have reduced skull density and aided their increasingly aquatic lifestyle [26,35,81,93,94], cancellous bone is still less buoyant than air-filled bones.

*Two*: reduction of braincase sinus infiltration may have been linked to changes in thalattosuchian feeding styles as they transitioned from land to water (i.e. increased muscle volume and the structural changes imposed on the skull). We propose that thalattosuchians, at least originally, were nearshore vision-based pursuit predators, as they had large and laterally oriented eye sockets [25,32] and lacked the sophisticated snout integumentary system [95] that aids prey detection in extant crocodylians given their poor underwater vision [96–99]. Given that water is a denser and more viscous medium than air, feeding primarily within the water column would have necessitated greater muscle contractile forces to aid in jaw closure. Thalattosuchians achieved this through the evolution of enormous supratemporal fenestrae, and by extension increased muscle volume [27,32,33,36,100–103]. In extant crocodylians, the bones of the braincase (quadrates, proötics, supraoccipital, basioccipital, basisphenoid and otoccipital) are pneumatized by the late embryonic phase (see [50,104]). Given that even juvenile thalattosuchians had large supratemporal fenestrae (albeit not as long, or in particular as wide, as adults [103,105]), it is possible that embryonic thalattosuchians had proportionally large temporal openings. If so, we propose a novel hypothesis: that the structural changes imposed on the embryonic skull by the enlarged temporal fenestrae (elongating the lateral and ventral elements of the braincase) imposed both mechanical constraints (the need for bony supports of the jaw musculature) and packaging constraints (less room into which air sinuses could expand) on the opportunistic growth of sinus diverticula. If so, this may have acted without any direct selection pressure to decrease buoyancy—an exaptation that improved diving performance, connecting these two hypotheses. We plan to investigate whether clades with elongated temporal fenestrae (= increased adductor musculature volume) show reduced pneumatic infiltration of the braincase bones in future studies.

*Three*: given their increasingly aquatic lifestyle, thalattosuchians had a decreased reliance on airborne sounds. The paratympanic sinuses contribute to the impedance-matching function of the middle ear, decreasing the stiffness of the middle ear and enhancing sensitivity to low-frequency sounds [50,88]. The mismatch in impedance is a major problem for terrestrial vertebrates relying on airborne sounds. A switch at the base of Thalattosuchia to sound reception underwater, which has very different acoustic properties, may have been a factor in sinus reduction. We hypothesize that when thalattosuchians no longer needed to have the impedance-matching of the middle ear to hear airborne sounds, the selection pressure to retain extensive paratympanic sinuses was no longer present. The lifting of this sense-related selection pressure would have made possible the regression of the paratympanic sinuses necessary for hypothesis one (increasing skull density). The intertympanic diverticulum aids directional hearing and helps acoustically couple the middle ears in extant archosaurs [50,86–89]. Thalattosuchians, like penguins, lost this sinus pathway [90]. Sørensen *et al.* [106] hypothesized that pressure gradient coupling of the two ears would not aid directional hearing underwater for penguins. If correct, then lack of a selection pressure to retain this sinus pathway may be another reason why the braincase sinuses regressed in thalattosuchians (and aquatic archosaurs more generally).

### 4.3. Metriorhynchids took sinus system evolution to the extreme

The pelagic metriorhynchids had the most transformed sinuses, of both the braincase and snout, as seen in Crocodylomorpha. They took thalattosuchian braincase sinus reduction to an extreme [41,42,44]. This is consistent with modern secondarily aquatic mammals—sea otters, pinnipeds, sirenians and cetaceans—that lack a frontal sinus, part of the paranasal system that infiltrates bones dorsal to the brain and is thus the functional equivalent of a portion of the paratympanic system in crocodylomorphs [11,85]. Most of these mammals, however, have completely lost bone-enclosed sinuses in their snout [11,107,108]. Metriorhynchids, on the other hand, possessed such sinuses [45–47]. The presence of snout sinuses in fully aquatic tetrapods is not unheard of, as the obligately pelagic basilosaurid-grade extinct cetaceans retained paranasal sinuses; later diverging cetaceans shed their bone-enclosed sinuses with these air-filled spaces either entirely within the soft tissue or only partly bound by the skull (‘extracranial sinuses’). This transformation in cetaceans possibly evolved to improve diving performance, with the extracranial sinuses later being incorporated into their sophisticated underwater sound production and reception systems [10–12,15–21]. Once unbound by the limitations of skull space, the extracranial pterygoid sinus system of cetaceans expanded, particularly in odontocetes and deep-diving species [10–12,15–17,21].

What is perhaps unexpected about metriorhynchids is that they increased the complexity and volume of their paranasal system relative to their ancestors, especially the presence of an extracranial air sac, the suborbital diverticula (i.e. the evagination of the most posterior portion of the antorbital sinus protruding into the orbit through the postnasal fenestra) [45]. Metriorhynchids also increased the size of the posterior antorbital cavity and the diameter of the postnasal fenestra at its posterior end [47]. It has been hypothesized that the paranasal sinus system of metriorhynchids may have been actively ventilated by jaw musculature contractions [109], as seen in extant birds and suggested for at least some non-avian theropods [54]. In metriorhynchids, this muscle-mediated active ventilation and inflation of the suborbital diverticula, and consequently the inflation of the antorbital sinus, could have compressed and helped drain the hypertrophied salt glands (see [109]). If so, these enormous glands (which need enough space to change their volume) may have constrained the sinuses from regressing. Interestingly, hypertrophied salt glands evolved before the suborbital diverticulum, as inferred from the semi-aquatic relative of metriorhynchids, *Eoneustes* [110]. We hypothesize that metriorhynchids evolved this mechanism for salt excretion as they became increasingly aquatic. Therefore, we posit that both cetaceans and metriorhynchids evolved extracranial sinuses due to different selection pressures.

The pelagic metriorhynchids are unusual in another aspect of their sinuses. Although their braincase sinuses were highly reduced, the middle ear cavity, which is confluent with the paratympanic sinus system [41,42,44], retained a small external auditory meatus [41,111]. Thus, they presumably had external ears with a tympanum. In many secondarily aquatic tetrapods, the middle ear chambers are enclosed by bone, such that they lack external ears, considered an adaptation for underwater hearing [112–114]. Transitional fossil cetaceans, including basilosaurids, retained an external auditory meatus, before more derived cetaceans closed off their middle and inner ear cavities and relied on a system in which the mandible and an accompanying fat pad conducted sound to the inner ear, not only bony ossicles associated with the meatus [112]. There is no sign from either braincase or mandible morphology that metriorhynchids were developing hearing systems analogous to cetaceans.

Metriorhynchids were a curious group. They evolved classic aquatic adaptations, such as a tail fin, flipper-like forelimbs, osteoporotic-like skeletal lightening and a smooth body integument (figure 1*c*; also see [27,28,33–37,81,82,93]). However, it took over 20 million years from when they first appeared in the fossil record for species with fully posterodorsally retracted external nares to evolve, their hind limbs remained large compared to their forelimbs (albeit with some paddle-like modifications), and they retained a pelvis–vertebral column articulation (figure 1*c*) [27,33,34,37,115–117]. When compared to cetaceans, metriorhynchids appear to have become ‘stuck’ in a ‘late protocetid-to-basilosaurid’ stage of aquatic specialization (e.g. [1,118,119]), unable to adapt further to an aquatic existence.

## 5. Conclusion

Thalattosuchians are one of several groups of tetrapods that left the land and began swimming and hunting in the water. They exhibit both similarities and differences compared to other secondarily aquatic groups, speaking to both general trends in how major evolutionary transitions unfold and peculiarities that have shaped each individual transition. The regression of braincase sinuses in thalattosuchians mirrored that of cetaceans: during their semi-aquatic phase sinuses generally reduced in size and complexity, and then diminished further as they became fully pelagic, probably for reasons related to buoyancy, diving and feeding [11–14,112]. However, once they reached a fully pelagic stage, both groups evolved extracranial sinuses. In cetaceans, these sinuses are an extension of the middle ear-related sinus system, whereas in metriorhynchids they are outgrowths of the nasal cavity-related sinus system. The cetacean extracranial pterygoid sinus system is thought to aid pressure regulation during deep dives and be involved with their sophisticated sound production and reflection abilities [11,12], while in metriorhynchids they have been hypothesized to have aided salt excretion [109]. In cetaceans, the caudodorsal retraction of the nares and the resulting generalized movement of the larynx potentially necessitated the loss of their maxillary and frontal paranasal sinuses [11], whereas metriorhynchids only started to show noticeable narial retraction in the Early Cretaceous (over 20 Ma after they evolved [115,117]). Because of pressure-related deformation, the expansive snout sinus system may have precluded metriorhynchids from diving particularly deeply, and therefore from exploring a wider range of ocean ecosystems despite the fact that they were past the point of no return to land.

In broad comparison, metriorhynchids appear to have reached the ‘late protocetid-to-basilosaurid’ grade of cetacean evolution: they were obligately pelagic with flippers, a tail fin, small braincase sinuses, retained some snout sinuses, evolved extracranial sinuses, and had a reduced external ear. Perhaps given more evolutionary time, and if not for their Early Cretaceous extinction, thalattosuchians may have gone further, and converged more thoroughly with modern cetaceans that can dive hundreds of metres deep. Or perhaps the need to mechanically drain their salt glands would have prohibited further aquatic specialization. In any case, the parallels and divergences between thalattosuchians and cetaceans show that major evolutionary transitions can traverse the same environmental endpoints and produce distantly related species that are convergent with each other in general terms but also constrained by their own particular anatomy, biology and evolutionary history.

## Data Availability

The online supplementary material, matrices, and R scripts can be found at the following link: https://doi.org/10.6084/m9.figshare.26270122.v1. The CT generated masks can be found at the following Morphosource pages. Project for the paper: https://www.morphosource.org/projects/000638758. Cricosaurus araucanensis (MLP 72-IV-7-1): https://www.morphosource.org/projects/000652928. Eoneustes gaudryi (NHMUK PV R 3353): https://www.morphosource.org/projects/000637139. 'Metriorhynchus' cf. brachyrhynchus (NHMUK PV OR 32618): https://www.morphosource.org/projects/000640896. Macrospondylus bollensis (NHMUK PV OR 14436): https://www.morphosource.org/projects/000652990. Macrospondylus bollensis (SNSB-BSPG 1984 1258): https://www.morphosource.org/projects/000652989. Pelagosaurus typus (NHMUK PV OR 32599): https://www.morphosource.org/projects/000637160. Plagiophthalmosuchus gracilirostris (NHMUK PV OR 15500): https://www.morphosource.org/projects/000652988. Plagiophthalmosuchus gracilirostris (NHMUK PV OR 33095): https://www.morphosource.org/projects/000638811. Plesiosuchus manselii (NHMUK PV R 1089): https://www.morphosource.org/projects/000652995. Thalattosuchus superciliosus (NHMUK PV R 11999): https://www.morphosource.org/projects/000638810. Torvoneustes coryphaeus (MJML K1863): https://www.morphosource.org/projects/000652117 https://www.morphosource.org/projects/000652117. Supplementary material is available online [120].
